# The anti-inflammatory role of extranuclear apurinic/apyrimidinic endonuclease 1/redox effector factor-1 in reactive astrocytes

**DOI:** 10.1186/s13041-016-0280-9

**Published:** 2016-12-16

**Authors:** Hyunjung Baek, Chae Seong Lim, Hee Sun Byun, Hyun Sil Cho, Yu Ran Lee, Yong Sup Shin, Hyun-Woo Kim, Byeong Hwa Jeon, Dong Woon Kim, Jinpyo Hong, Gang Min Hur, Jin Bong Park

**Affiliations:** 1 Department of Physiology and Department of Medical Science, School of Medicine, Chungnam National University, 266 Munhwa-Ro, Jung-gu, Daejeon, 30501 Republic of Korea; 2 Department of Anesthesiology & Pain Medicine, School of Medicine, Chungnam National University, Daejeon, 30501 Republic of Korea; 3 Department of Pharmacology, School of Medicine, Chungnam National University, Daejeon, 30501 Republic of Korea; 4 Department of Anatomy and Department of Medical Science, School of Medicine, Chungnam National University, Daejeon, 30501 Republic of Korea

**Keywords:** APE1/Ref-1, Astrocytes, Inflammation, iNOS, TNF-α

## Abstract

Apurinic/apyrimidinic endonuclease 1 (APE1), a ubiquitous multipurpose protein, is also known as redox effector factor-1 (Ref-1). It is involved in DNA repair and redox signaling and, in turn, oxidative stress-induced neurodegeneration. Although previous studies have demonstrated that APE1/Ref-1 functions as a negative regulator of inflammatory response via several mechanisms in neuronal cells, little is known about the roles of APE1/Ref-1 in glial cells. In this study, we found that cytoplasmic APE1/Ref-1 expression was upregulated in reactive astrocytes of the kainic acid- or lipopolysaccharide (LPS)-injected hippocampus. Analysis of the inflammatory response induced by extranuclear APE1/Ref-1 (ΔNLS-Ref-1) in cultured primary astrocytes revealed that it markedly suppressed inducible nitric oxide synthase (iNOS) expression and tumor necrosis factor-α (TNF-α) secretion induced by LPS to a similar extent as did wild type APE1/Ref-1 (WT-Ref-1), supporting the concept an anti-inflammatory role of extranuclear APE1/Ref-1 in astrocytes. Additionally, overexpression of WT- and ΔNLS-Ref-1 suppressed the transcriptional activity of nuclear factor-κB (NF-κB), although it effectively enhanced activator protein 1 (AP-1) activity. The blunting effect of APE1/Ref-1 on LPS-induced NF-κB activation was not mediated by IκB kinase (IKK) activity. Instead, APE1/Ref-1 inhibited p300-mediated acetylation of p65 by suppressing intracellular reactive oxygen species (ROS) levels following LPS treatment. Taken together, our results showed that altered expression and/or subcellular distribution of APE1/Ref-1 in activated astrocytes regulated the neuroinflammatory response to excitotoxin and endotoxin insults used in model of neurodegenerative brain diseases.

## Introduction

Apurinic/apyrimidinic endonuclease 1 (APE1), a ubiquitous multipurpose nuclear protein, is involved in the base excision repair pathway for damaged bases and DNA single-strand breaks following endogenous and exogenous oxidative stress. APE1 acts as a reductive activator of many transcription factors involved in apoptosis, inflammation, angiogenesis and survival pathways [1–4], and also known as redox effector factor-1 (Ref-1). APE1/Ref-1 is highly expressed in vivo in specific brain regions, such as the hippocampus and cerebral cortex [5, 6]. It plays a neuroprotective role in brain pathology characterized by increased inflammation and oxidative stress, such as ischemic [7, 8] or compression injury [9] and neurodegeneration [10–12]. Astrocytes, the most numerous non-neuronal cell type, comprise ~50% of human brain volume [13], and express high levels of APE1/Ref-1. However, despite the wealth of information available on neuronal APE1/Ref-1 in brain diseases, the functional significance of APE1/Ref-1 in glial cells is unclear.

In addition to its classical role as a nuclear protein, extranuclear APE1/Ref-1 controls the intracellular redox state by inhibiting reactive oxygen species (ROS) production via negative regulation of the activity of Rac1, a Ras-related GTPase [14]. The cytoplasmic/nuclear distribution appears to fine tune the anti-inflammatory activity of APE1/Ref-1 [15, 16]. Astrocytes are important sources of proinflammatory mediators, such as inducible nitric oxide synthase (iNOS) and tumor necrosis factor-α (TNF-α), which modulate various brain pathophysiologies [17–19]. Astrocytic APE1/Ref-1, especially extranuclear APE1/Ref-1, may regulate neuroinflammatory process in the brain. Here we show that cytoplasmic APE1/Ref-1 inhibited the iNOS expression and TNF-α secretion of reactive astrocytes in the excitotoxin- and endotoxin-challenged brain, at least in part by negatively regulating ROS and NF-κB signaling.

## Results

### Changes in APE1/Ref-1 expression and subcellular translocation in reactive astrocytes

Previous studies demonstrated that APE1/Ref-1 expression was increased in both surviving and vulnerable neurons following inflammatory insults [7, 20]. To assess whether this is also the case in reactive astrocytes, we investigated APE1/Ref-1 expression in astrocytes from kainic acid (KA)- or lipopolysaccharide (LPS)-treated brains.

**Fig. 1:**
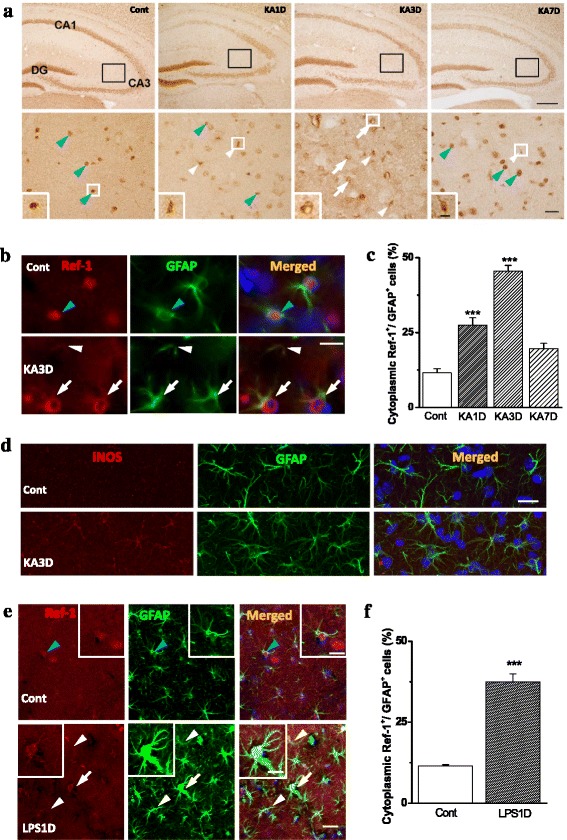
Altered APE1/Ref-1 expression in the kainic acid (KA)-injured hippocampus. **a** APE1/Ref-1 immunoreactivity in the hippocampus at 1, 3, and 7 d after KA injection. Higher magnification of areas in CA3 region of the hippocampus showed sequential changes of APE1/Ref-1 expression (*lower* panel; scale bar = 20 μm). Note that APE1/Ref-1-ir appeared in small nuclei and short processes (glial morphology, *white arrowheads*), or faint nuclei and dense cytoplasm (*white arrows*) in KA treated hippocampus, while it mostly appeared in the round nuclei in control brain (*blue arrowheads*). **b** Representative photomicrographs from the double immunofluorescence staining for APE1/Ref-1 (*red*) and GFAP (*green*) in the CA3 region of KA-treated hippocampi (3 d after KA injection). APE1/Ref-1 was found in the processes (*white arrowheads*) and perinuclear cytoplasm (*white arrows*) of GFAP-positive astrocytes. Nuclei were counterstained with DAPI (*blue*). **c** Quantitative analysis of double immunofluorescence staining for the identification of APE1/Ref-1-positive cells in the KA-injured hippocampus. The portion of APE1/Ref-1-positive cells out of total glial fibrillary acidic protein (GFAP)-positive cells was compared at 1, 3, and 7 d in the KA-injured hippocampus. ***P ˂ 0.001 vs. saline-injected control. **d** Representative photomicrographs showing that GFAP-positive cells expressed iNOS in the KA-treated groups. Nuclei were counterstained with DAPI (*blue*). **e** Representative photomicrographs showing that APE1/Ref-1 was expressed in reactive astrocytes in the LPS-treated hippocampi (1 d after LPS injection). APE1/Ref-1 was found in the processes and cytoplasm of GFAP-positive astrocytes (*white arrowheads*). Nuclei were counterstained with DAPI (*blue*). Scale bar = 200 μm (or 20 μm, in set). **f** Quantitative analysis of double immunofluorescence staining for the identification of APE1/Ref-1-positive cells in the LPS-injured hippocampus. ***P ˂ 0.05 vs. saline-injected control

KA treatment resulted in a clear pattern of behavioral seizures that began within 20 min after the injection, and progressed to tonic-clonic activity. KA-induced excitotoxicity has been used as a model for examining mechanisms underlying oxidative stress and inflammation. Thus, APE1/Ref-1 expression was compared in control and KA-treated brains. APE1/Ref-1-immunoreactivity (ir) consisted of nuclei from neurons and astrocytes in KA-vulnerable regions, including the hippocampal principle neuronal layers and hilar sub-regions. While most APE1/Ref-1-ir was in neurons in both the control and KA-treated groups, APE1/Ref-1 positive cells exhibited glial morphology with small nuclei and short processes, or faintly stained nuclei and dense cytoplasm, especially in hilar region of KA-treated brains (Fig. 1a). Double immunofluorescence staining showed that APE1/Ref-1-ir was increased in glial fibrillary acidic protein (GFAP)-positive cells in the CA3 region of KA-treated hippocampi. APE1/Ref-1-positive astrocytes increased at 1 d post-lesion, became maximal at 3 d, and recovered to control level by 7 d after KA-injection (Fig. 1b). Furthermore, APE1/Ref1-ir was found in the processes and cytoplasm of astrocytes (Fig. 1c). The Western blot analysis showed that there was a tendency for APE1/Ref-1 levels to increase in the whole hippocampus in KA-treated groups (data not shown), but this did not reach statistical significance at 1-7 d after KA-injection (*p* > 0.1). Double immunofluorescence staining showed that reactive astrocytes expressed iNOS in the KA-treated hippocampus (Fig. 1d). These results indicated that cytoplasmic APE1/Ref-1 expression was upregulated in reactive astrocytes under KA-induced oxidative stress and inflammatory conditions.

To assess if our observation was generalizable, we also examined the subcellular distribution of APE1/Ref-1 expression in an LPS-challenged in vivo model. Cytoplasmic translocation of APE1/Ref-1 in astrocytes was also observed in the LPS-treated brain. APE1/Ref-1-ir was clearly detected in the processes and cytoplasm of reactive astrocytes after LPS treatment (Fig. 1e), which increased significantly in the CA3 regions of the LPS-treated hippocampus (Fig. 1f).

### Ectopic expression of APE1/Ref-1 abrogates inflammatory responses in primary cultured astrocytes

To investigate whether APE1/Ref-1 is involved in the inflammatory response, primary cultured astrocytes that showed high APE1/Ref-1 expression in in vivo experiments above were infected with AdRef-1.

**Fig. 2:**
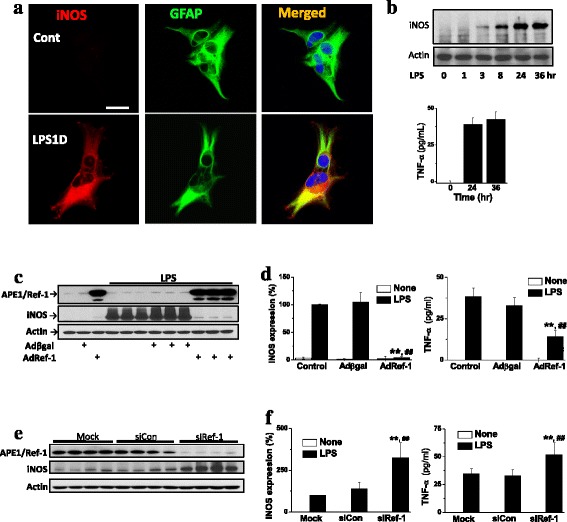
APE1/Ref-1 inhibits LPS-induced iNOS expression and TNF-α secretion in primary cultured astrocytes. **a** Representative photomicrographs showing that LPS treatment induced iNOS expression in cultured astrocytes. Scale bar = 20 μm. **b** LPS increases iNOS expression and TNF-α secretion from cultured astrocytes in a time-dependent manner. Cells and media were harvested after treatment with LPS (100 nM) for the indicated times. **c** Western blot analysis for APE1/Ref-1 and iNOS was performed at 24 h on LPS-treated cells. Ectopic expression of APE1/Ref-1 using APE1/Ref-1 adenovirus (AdRef-1) inhibited LPS-stimulated iNOS expression. **d** Summarized data showing that APE1/Ref-1 inhibited LPS-induced iNOS expression (left) and TNF-α secretion (right). Data represent the mean ± standard error of mean (SEM; *n* = 4). ** *p* < 0.01 vs. non-transfected control cells; ^##^ *p* < 0.01 vs. β-galactosidase virus (Adβgal)-transfected control cells by a two-way anaysis of variance (ANOVA) followed by Dunnett’s test. **e** Reduced APE1/Ref-1 expression by short interfering RNA (siRNA; 100 pmol) significantly enhanced LPS-induced iNOS expression. **f** Summarized data showing that siRNA for APE1/Ref-1 (siRef-1) increased LPS-induced iNOS expression (left) and induced TNF-α secretion (right) in astrocytes. Data represent the mean ± SEM of four separate experiments. **, ^##^ *p* < 0.01 vs. non-transfected control cells and scrambled RNA (siCon) by two-way ANOVA, followed by Dunnett’s test, respectively

In agreement with previous reports [21–24], LPS induced iNOS expression in cultured astrocytes (Fig. 2a). LPS treatment resulted in a rapid and progressive induction of iNOS expression and TNF-α production in a time-dependent manner, while the basal levels of these inflammatory mediators were barely detectable in control cells (Fig. 2b), indicating that inflammatory reactions can be triggered effectively in this primary cells. Remarkably, overexpression of APE1/Ref-1 almost completely blocked LPS-induced iNOS expression and TNF-α production (Fig. 2c, d). This indicated that APE1/Ref-1 was able to downregulate the LPS-induced inflammatory response in primary astrocytes.

To confirm the role of endogenous APE1/Ref-1 against the inflammatory response to LPS, we next examined the extent of LPS-induced iNOS and TNF-α production in APE1/Ref-1 knocked-down primary astrocytes using specific small interfering RNA (siRNA). Consistently, APE1/Ref-1 knockdown resulted in significantly enhanced iNOS expression and TNF-α production induced by LPS, versus that in cells treated with scrambled RNA or uninfected control cells (Fig. 2e, f). These results suggest that APE1/Ref-1 has the capacity to downregulate the LPS-induced inflammatory response in reactive astrocytes.

### Role of cytoplasmic APE1/Ref-1 against the LPS-induced inflammatory response in primary cultured astrocytes

Certain conditions, including inflammation, lead to APE1/Ref-1 translocation from the nucleus into the cytoplasm, which is critical for APE/Ref-1 activity [25]. Having shown that increased APE1/Ref-1 was localized in the cytoplasm of reactive astrocytes in both KA- and LPS-challenged brains (Fig. 1), we next examined the functional significance of cytoplasmic APE1/Ref-1 in astrocytes activated by inflammatory insults. To address this, we generated and transiently transfected a mutant construct of APE1/Ref-1 (ΔNLS-Ref-1), which lacked the N-terminal nuclear import sequence (NLS), in primary cultured astrocytes. Consistent with our previous report [16], ΔNLS-Ref-1 was localized exclusively in the cytoplasm, while the majority of wild-type APE1/Ref-1 (WT-Ref-1) expression was in the nucleus (data not shown).

**Fig. 3:**
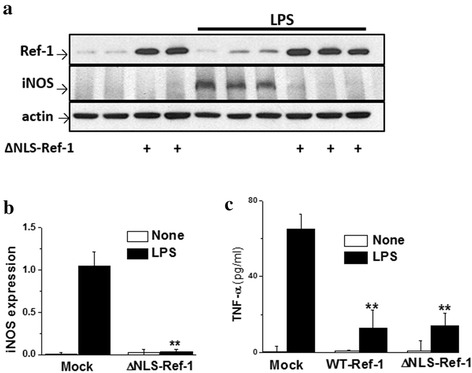
N-terminus deletion mutant APE1/Ref-1 (ΔNLS-Ref-1) inhibits LPS-induced iNOS expression and TNF-α secretion in astrocytes. **a** Western blot analysis showing the effects of ΔNLS-Ref-1 expression by plasmid transfection on LPS-induced iNOS expression. **b** Summarized data showing the ΔNLS-Ref-1 inhibition of LPS-induced iNOS expression, as in A. Data represent the mean ± SEM (*n* = 4). **c** Effects of wild-type (WT-Ref-1) and ΔNLS-Ref-1 overexpression on LPS-induced TNF-α secretion from astrocytes. Data represent the mean ± SEM of three separate experiments. ** *p* < 0.01 vs. non-transfected control cells

LPS-induced iNOS expressions was markedly suppressed in ΔNLS-Ref-1-transfected astrocytes (Fig. 3a, b). Consistent with this, ΔNLS-Ref-1 was able to suppress LPS-induced TNF-α secretion to a degree similar to that obtained with WT-Ref-1 (Fig. 3c). Combined with the enhanced expression of cytoplasmic APE1/Ref-1 in the proinflammatory injured brain, these observations suggest that, in the cytoplasm, APE1/Ref-1 functions as an important negative regulator to suppress inflammatory reactions.

### Extra-nuclear APE1/Ref-1 acts as a negative regulator of NF-κB signaling without affecting IκB kinase activity

It has been established that NF-**κ**B activation is an essential process for the transcriptional induction of proinflammatory cytokines including iNOS and TNF-α. To determine the molecular mechanism involved in the APE1/ref-mediated anti-inflammatory response, we next examined whether the expression levels of APE1/Ref-1 affect the transcriptional activity of NF-κB.

**Fig. 4:**
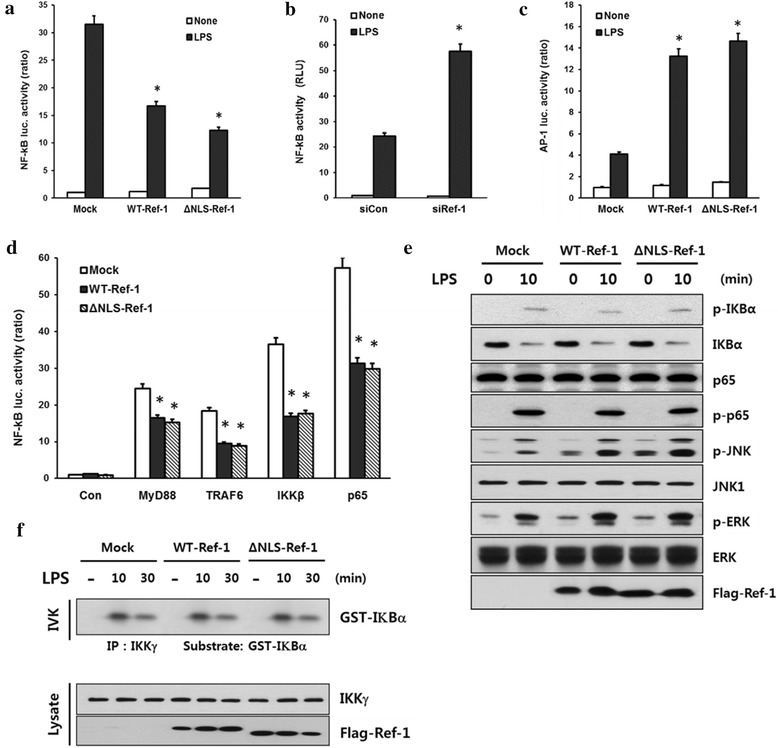
APE1/Ref-1 regulates LPS-mediated NF-κB signaling without affecting IKK activity. **a**, **b** Primary cultured astrocytes were transfected with p2xNF-κB-Luc and pRSV-β-gal with expression plasmids for the flag-tagged APE1/Ref-1 (wild-type- and ΔNLS-Ref-1) or APE1/Ref-1 short interfering RNA (siRef-1; 100 pmol). **c** Primary cultured astrocytes were transfected with the expression plasmids of the p2xTRE-Luc, pRSV-β-gal, WT-, and ΔNLS-Ref-1. After 24 h of transfection, cells were treated with LPS (1 μg/mL) for 6 h. **d** 293/TLR4/IL-1R cells were transiently transfected with the expression plasmids indicated, along with p2xNF-κB-Luc and pRSV-β-gal. Luciferase assays were performed as described in the Methods, and the activity of each sample was normalized according to the β-galactosidase activity. Each column shows the mean ± SEM of at least three independent experiments. *p ˂ 0.05 vs. mock-transfected cells. **e**, **f** 293/TLR4/IL-1R cells were transiently transfected with either wild-type- or ΔNLS-Ref-1 for 24 h, and then cells were treated with LPS for the times indicated. Whole-cell lysates were immunoblotted with the indicated antibodies (**e**). Cell extracts were subjected to immunoprecipitation with an anti-IKK-γ antibody, and its activity was assessed by an immune complex kinase assay, as described in the Methods (**f**, *upper* panel). The specificity of the immunoprecipitation of the IKK complex was confirmed by immunoblotting with an anti-IKK-γ antibody (**f**, *lower* panel)

Ectopic overexpression of APE1/Ref-1 (WT-Ref-1 and ΔNLS-Ref-1) resulted in a significant decrease of NF-κB activity in response to LPS (Fig. 4a). Consistently, APE1/Ref-1 knockdown resulted in a marked increase in NF-κB reporter activity induced by LPS (Fig. 4b). In contrast to anti-NF-κB properties of APE1/Ref-1, LPS-induced AP-1 activation was significantly enhanced by overexpression of the wild-type and ΔNLS forms of APE1/Ref-1 (Fig. 4c). This indicate that ΔNLS-Ref-1 was able to downregulate NF-κB signaling to a similar extent as did WT-Ref-1, whereas it upregulated AP-1 transcriptional activity. Confirming this, NF-κB activation, mediated by the NF-κB component (p65), IκB kinase β (IKKβ) and adaptor proteins (MyD88, TRAF6) was also significantly attenuated by ΔNLS-Ref-1 in 293/TLR4/IL-1R cells stably expressing TLR4 and IL-1R (Fig. 4d).

To gain insight into the specificity of the molecular mechanisms underlying NF-κB regulation by APE1/Ref-1, we further analyzed the IKK-mediated phosphorylation/degradation of IκBα. Unexpectedly, wild-type- and ΔNLS-Ref-1 failed to downregulate the phosphorylation and degradation of IκBα, whereas both types of APE1/Ref-1 were able to enhance the phosphorylation of c-jun N-terminal kinase (JNK) and extracellular signal-regulated kinase (ERK) in response to LPS (Fig. 4e). These results indicated that downregulation of LPS-induced NF-κB activation by Ref-1 was independent of IKK. Consistently, LPS-induced IKK activity was not affected by wild-type- or ΔNLS-Ref-1 overexpression (Fig. 4f), confirming that APE1/Ref-1 likely functions downstream of IKK signaling components in downregulating NF-κB transcriptional activity.

### Ref-1 abolishes p300-mediated p65 acetylation via suppression of cellular ROS levels

**Fig. 5:**
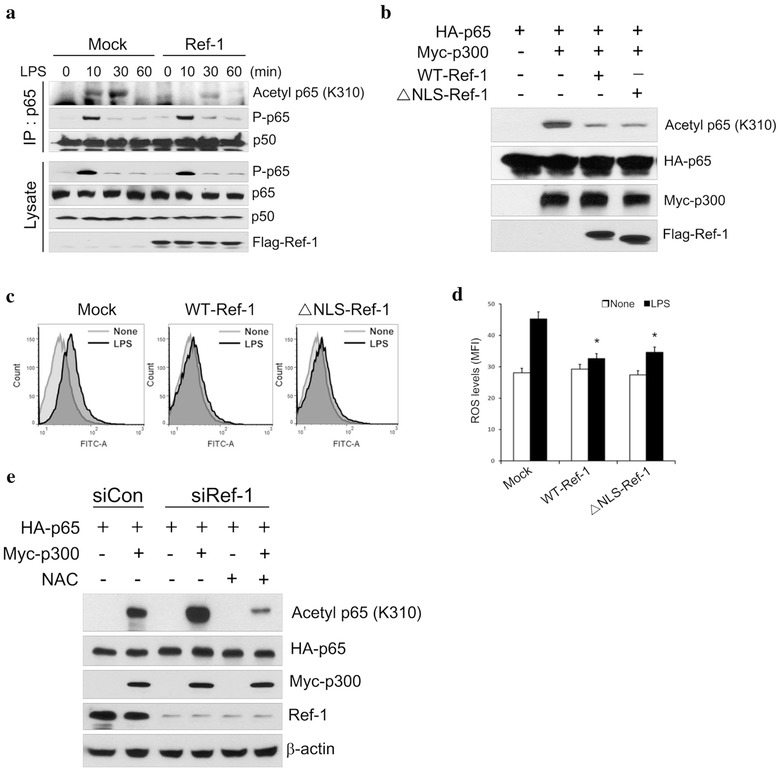
APE1/Ref-1 suppresses p300-mediated p65 acetylation via suppressing cellular ROS levels. **a** Primary cultured astrocytes were transiently transfected with wild-type- or ΔNLS-Ref-1, followed by LPS treatment for the times indicated. After immunoprecipitation with an anti-p65 antibody, the acetylation levels of p65 were detected by immunoblotting with an acetyl-lysine-specific antibody (top row). Co-immunoprecipitated p50 served as a positive control (top second row). Whole-cell lysates were immunoblotted with the antibodies indicated. **b** 293/TLR4/IL-1R cells were co-transfected with expression plasmids HA-p65 and Myc-p300 along with flag-tagged wild-type- or ΔNLS-Ref-1. Whole cell lysates were subjected to immunoblotting with anti-acetyl-p65, HA, Myc, and flag antibodies. **c** Primary cultured astrocytes were transiently transfected with either wild-type- or ΔNLS-Ref-1, as described in (**a**), followed by LPS treatment for 10 min. ROS levels were measured with a flow cytometer (*top* panel) as described in the Methods. **d** Data were processed and quantified with FlowJo software. **e** 293/TLR4/IL-1R cells were transfected with either 100 pmol of scramble (control; siCon) or APE1/Ref-1 short interfering RNA (SiRef-1) along with the expression plasmids HA-p65 and Myc-p300 in the absence or presence of NAC (10 mM). Whole-cell lysates were subjected to immunoblotting with the indicated antibodies

Because it seemed that the inhibitory mechanism of APE1/Ref-1 on LPS-induced NF-κB activation was not associated with IKK activity, we next examined whether APE1/Ref-1 affected the acetylation status of the p65 subunit of NF-κB, an important event in the transcriptional activation of NF-κB. To address this, the primary cultured astrocytes were treated with LPS for different time points, and cell extracts were collected for immunoprecipitation experiments with anti-p65 antibodies. We found that endogenous acetylation of p65 was detectable in the immunoprecipitates, peaking at 30-min post LPS treatment (Fig. 5a, top panel, left). This LPS-induced acetylation of p65 was abolished in APE1/Ref-1 overexpressing cells without altering phosphorylation levels, even though comparable amounts of total p50 were immunoprecipitates in each sample (Fig. 5a, top to third panel). These results suggest that the inhibitory potency of APE1/Ref-1 with regard to p65 acetylation contributes to downregulating the LPS-induced NF-κB response.

To confirm these observations, we compared the ability of wild-type- and ΔNLS-Ref-1 to regulate p65 acetylation induced by the overexpression of p300 acetyltransferase, which has an essential role in p65 acetylation through a physical interaction. Consistent with previous reports [26, 27], overexpression of p300 resulted in a remarked increase in p65 acetylation, and this p300-mediated acetylation was almost completely impaired in wild-type- and ΔNLS-Ref-1-transfected cells (Fig. 5b, top panel). This suggested that extranuclear APE1/Ref-1 interrupted p300-mediated p65 acetylation, but at early stage of IKK-mediated p65 phosphorylation.

It has been established that the catalytic activity of p300 in human cells is directly regulated by the cellular redox state [28, 29]. Furthermore, convincing evidence indicates that APE1/Ref-1 controls the level of intracellular ROS, which has been linked to the regulation of many transcription factors. Therefore, we hypothesized that the inhibitory effects of wild-type- or ΔNLS-Ref-1 on LPS-induced p65 acetylation might be achieved through regulation of intracellular ROS levels. As expected, overexpression of ΔNLS-Ref-1 significantly blunted LPS-induced ROS production to a similar extent as did wild type-Ref-1 (Fig. 5c, d). Furthermore, treatment with the ROS scavenger N-acetyl-L-cysteine (NAC), markedly suppressed p300-mediated p65 acetylation under conditions of ectopic expression of ΔNLS-Ref-1 or knocked-down expression of APE1/Ref-1 (Fig. 5e). Therefore, these findings suggest that cytoplasmic APE1/Ref-1 is able to effectively suppress LPS-induced p65 acetylation via its antioxidant role, acting as a negative regulator of NF-κB signaling.

## Discussion

The main findings of the present study were as follows: 1) APE1/Ref-1 expression with cytoplasmic translocation was increased in reactive astrocytes in hippocampi treated with KA or LPS; 2) both wild-type APE1/Ref-1 and ΔNLS-Ref-1 acted as negative regulators of the inflammatory response in primary cultured astrocytes, while APE1/Ref-1 knockdown significantly enhanced the inflammatory response; and 3) WT- and ΔNLS-Ref-1 blunted p300-mediated p65 acetylation without altering the phosphorylation status via their antioxidant properties, leading to NF-κB inhibition. Our findings provide the first evidence that cytoplasmic APE1/Ref-1 regulates excitotoxin- and endotoxin-induced neuroinflammatory events in brain reactive astrocytes via NF-κB dependent pathway.

While APE1/Ref-1 is highly expressed in proliferating cells during embryonic and early postnatal development in the hippocampus and piriform cortex [30, 31], APE1/Ref-1 expressed to a less extent in the adult brain, particularly in astrocytes [5]. In the present study, the principle neuronal layers of the hippocampus consistently showed much higher immunoreactivity than the surrounding areas in the normal brain (Fig. 1a). However, we observed the significantly enhanced APE1/Ref-1 expression in the cytoplasm of proinflammatory mediator-induced reactive astrocytes in brains injured with KA or LPS. Furthermore, overexpression of extranuclear APE1/Ref-1 blocked the LPS-induced inflammatory response in primary cultured astrocytes. Thus, enhanced cytoplasmic expression of APE1/Ref-1 in reactive astrocytes may act as a negative regulator to prevent excessive inflammatory reactions such as iNOS expression and TNF-α secretion.

APE1/Ref-1 may play a role in immunocompetent cells of the brain during aluminum chloride (AlCl_3_)-induced neuroinflammation, which could be prevented by treatment with resveratrol [32]. When resveratrol reversed AlCl_3_-induced neuroinflammation, increased APE1/Ref-1 coincide with reduced expression of TNF-α, IL-6 and iNOS in the brain. Combined with the finding that AlCl_3_ induced the production of proinflammatory cytokines and NO in both microglia and astrocytes [33], Zaky et al. suggested a role for glial APE1/Ref-1 in the resveratrol-mediated inhibition of neuroinflammation [32]. However, the neuronal affects were not differentiated from the glial effects in the resveratrol-induced anti-inflammatory response. In the current study, we provide direct evidence that increased cytoplasmic APE1/Ref-1 contributes to anti-inflammatory processes in reactive astrocytes during neuroinflammation in the brain.

While the expression of APE1 is primarily nuclear, APE1/Ref-1 undergoes active shuttling between the cytoplasm and the nucleus depending on the cell type and certain pathological conditions, including oxidative stress and inflammation [14, 34, 35]. Although the subcellular localization of APE1/Ref-1 is commonly associated with the redox state of the cysteine residues of APE1/Ref-1 [16, 36], the functional role of APE1/Ref-1 in the inflammatory process varies by cell types. For example, the cytoplasmic localization of APE1/Ref-1 was associated with its anti-Inflammatory activity in monocytes and endothelial cells [37, 38]. In contrast, pharmacological inhibition of APE1/Ref-1 suppressed the inflammatory response in activated macrophages [39]. The cytoplasmic APE1/Ref-1 localization mediating an anti-inflammatory response is consistent with ROS inhibition in endothelial cells [37, 38], while the translocation of APE1/Ref-1 from the cytoplasm to nucleus has been observed with various redox-related stimuli [35, 40]. In the present study, the prominent cytoplasmic APE1/Ref-1-ir in vivo was detected only in reactive astrocytes (Fig. 1), suggesting a possible role of APE1/Ref-1 during the neuroinflammatory process in the injured brain. Furthermore, our results showing that cytoplasmic APE1/Ref-1, overexpressed with ΔNLS-Ref-1, mimicking the anti-inflammatory role of wild-type APE1/Ref-1 overexpression in LPS-stimulated cultured astrocytes, are consistent with cytoplasmic APE1/Ref-1 effectively mediating the anti-inflammatory response during oxidative stress.

Our results showing that APE1/Ref-1 knockdown resulted in significantly enhanced iNOS expression and TNF-α production induced by LPS further suggested that endogenous APE1/Ref-1 paly an anti-inflammatory role in reactive astrocytes. Given that oxidative stress and inflammation could elicit either the up-regulation of expression or the cytoplasmic translocation of APE1/Ref-1, cytoplasmic translocation of APE1/Ref-1 may not be essentially depend on the protein synthesis in reactive astrocytes. Indeed, LPS induced cytoplasmic translocation of APE1/Ref-1 in cultured astrocytes with minimal changes in APE/1Ref-1 expression in the present study (Fig. 2). Similarly, in our previous report [16], TNF-α induced subcellular translocation rather than *de novo* protein synthesis to increase cytoplasmic APE1/Ref-1 in endothelial cells. However, cellular mechanism(s) for the cytoplasmic up-regulation of APE1/Ref-1 in inflamed brain is an open question to be further studied. Furthermore, iNOS expression in reactive astrocytes in KA-injured brain (Fig. 1) suggested that cytoplasmic APE1/Ref-1 translocation was not enough to completely abolish iNOS expression in the brain injury. Given that various resident and infiltrated inflammatory cells involve in the inflamed brain [41], APE1/Ref-1 shRNA delivery in specific glial cells would dissect the anti-inflammatory role of cytoplasmic APE1/Ref-1 in reactive astrocytes among others.

An important finding of this study was that extranuclear APE1/Ref-1 inhibited the transcriptional activity of NF-κB, even though it efficiently enhanced AP-1 activity. Earlier biochemical studies revealed that APE1/Ref-1 directly interacted with and reduced p50, a NF-κB subunit, leading to enhanced DNA-binding activity [42, 43]. However, due to lack of APE1/Ref-1-deficient mice, the exact role of APE1/Ref-1 in NF-κB signaling cascade in different cells or tissues remains unclear. The redox status in cells plays an essential role in NF-κB activation and nuclear chromatin remodeling (acetylation vs. deacetylation). Thus, one possibility of the discrepancy with previous report is that, the capacity of APE1/Ref-1 to act as a redox chaperone might be predominantly involved in downregulation of NF-κB transcriptional activity in response to LPS in cells harboring abundant NF-κB signaling machinery such as primary cultured astrocytes or 293/TLR4/IL-1R cells. This interpretation is supported by the observations that extranuclear APE1/Ref-1 suppresses LPS-induced ROS production and p65 acetylation to a similar extent as wild-type APE1/Ref-1, without altering IKK activity. Importantly, we found that such p300-mediated p65 acetylation was masked by overexpression of both types of APE1/Ref-1 and with the antioxidant NAC. These findings indicate that APE1/Ref-1 ensures effective shutdown of p65 acetylation via modulating intracellular ROS levels within the nucleus and cytoplasm, suppressing NF-κB transcriptional activity. Nevertheless, how APE1/Ref-1 controls and regulates the p65 acetylation is a question that remains largely unanswered.

Astrocytes are the main glial cell type in the brain involved in maintaining CNS homeostasis, and respond promptly to injury and regulate neuroinflammatory events [44, 45]. Both in vitro and in vivo studies have documented the ability of astrocytes to produce various cytokines and chemokines. Proinflammatory cytokines released from activated glial cells may lead to neuronal death, causing neuropathological changes in central nervous system (CNS) diseases, such as multiple sclerosis [46, 47], Parkinson’s disease (PD) [48, 49] and Alzheimer’s disease (AD) [50]. Our results suggest that extranuclear APE1/Ref-1 inhibits proinflammatory mediators, including iNOS and TNF-α in astrocytes. Therefore, limiting inflammatory cytokine production in reactive astrocytes via APE1/Ref-1 activity expected to be beneficial for the prevention of neuroinflammation and neurodegeneration in various brain diseases.

In neurodegenerative diseases involving oxidative DNA damage, such as AD, PD, and amyotrophic lateral sclerosis (ALS), decreased neuronal expression of APE1/Ref-1 after neuronal insult decreases cell viability and promotes neurodegeneration [51]. In addition to the expression of APE1/Ref-1, alterations in its subcellular localization reportedly play a role in neurodegenerative disease. Given that a seizure insult increases oxidative stress and inflammatory process [52], it is not surprising that APE1/Ref-1 activity is altered in the epileptic hippocampus. Indeed, our results showing that a KA-injection reduced APE1/Ref-1-ir in pyramidal cell layers (Fig. 1a) is consistent with a previous report [53]. Our results also showed that extranuclear translocation of APE1/Ref-1 was increased in astrocytes of the excitotoxin- and endotoxin-injured brain, which would reduce the neuroinflammatory response. To our knowledge, this is the first reported evidence that the subcellular translocation of APE1/Ref-1 in astrocytes is involved in neuroinflammatory events in the brain diseases, whereas neuronal APE1/Ref-1 has been linked with various brain oxidative stresses.

Overall, our results are the first reported evidence that altered subcellular translocation of APE1/Ref-1 in reactive astrocytes mediates anti-inflammatory events, thus, highlighting a new pathway involved in regulating inflammatory brain disease.

## Methods

### Animals

Animals housed under a 12/12-h light/dark schedule had access to food water ad libitum throughout the experiments. All animal experiments adhered to the Chungnam National University policies regarding the care and use of animals. As described previously [54, 55], KA was injected using a 50-μl Hamilton microsyringe fitted with a 26-gauge needle (0.1 μg/5 μl in PBS, i.c.v), and LPS was injected intraperitoneally (10 mg/kg, i.p.). The same volume of phosphate-buffered saline was injected i.c.v or i.p. as a respective control.

### Immuonohistochemistry and double Immunofluorescence

Sections (25 μm) from post-fixed brains in 4% paraformaldehyde were cut using a cryostat as described previously [56]. Free-floating sections subjected to immunohistochemical staining using the avidin-biotin peroxidase complex (ABC) method were incubated with primary anti-APE1/Ref-1 (1:200 dilution). Double immunohistochemical fluorescent reactions were used to study the expression of APE1/Ref-1 or iNOS, and their possible colocalization with astroglial cell (glial fibrillary acid protein, GFAP immunoreactive). Sections were incubated for 24 hs in the presence of a polyclonal rabbit anti APE1/Ref-1 primary antibody in conjunction with a monoclonal mouse anti GFAP (1:10,000 dilution) antibody. Incubation in primary antibodies were followed by the incubation in secondary antibodies (donkey anti-rabbit Cy3 labeled and donkey anti-mouse FITC labeled, 1:200, respectively). Nucleus staining was performed with DAPI. Digital microscopy was performed with a Zeiss Axioscope microscope equipped with a digital CCD camera (AxioCam and AxioVision 4.8 software, Zeiss, Germany) or Leica confocal microscope (Leica-Microsystems, Germany).

A positive co-localization was considered by the appeared yellow (red + green) profiles in merged images, similar in size, shape and geometry in red and green profiles.

### Cells, immunoblot analysis and immunoprecipitation

Primary astrocytes were cultured from the neonatal rats, as described previously [57]. In brief, removed brain cortices were triturated in minimal essential medium (MEM; Sigma, St. Louis, MO, USA) containing 10% fetal bovine serum (FBS; HyClone, Logan, UT, USA), plated in 75 cm^2^ T-flasks, and incubated for 2–3 weeks. Microglia were detached from flasks by mild shaking, and primary astrocytes remaining in the flask were harvested with 0.1% trypsin. Astrocytes were plated in 100-mm dishes, and cultured in MEM containing 10% FBS.

Primary astrocytes and a human embryonic kidney (HEK) 293 cells stably expressing the IL-1 receptor and toll-like receptor 4 (293/IL-1R/TLR4) were maintained at 37 °C in a humidified incubator contain 5% CO_2_. For the knockdown experiments, cells were transfected with 50 pmol of rat APE1/Ref-1 or human APE1/Ref-1 (Bioneer, South Korea, 1006541)-specific siRNA (siRef-1) using Lipofectamine 2000 reagent (Invitrogen) according to manufacturer’s instruction. For the ectopic expression, cells were transfected with wild-type APE1/Ref-1 (WT-Ref-1) or ΔNLS-Ref-1 in DsRed expression vector [58]. ΔNLS-Ref-1 encodes APE1/Ref-1 with a 28-aa deletion of the putative N-terminal nuclear localization signal [16]. Alternatively, cells were infected with recombinant virus encoding β-galactosidase (Adβgal) or full-length APE1/Ref-1 (AdRef-1) with the titers of 200 PFU per cells [58].

After treatment as described in the figure legends, cells were collected and lysed. Cell lysates were fractionated by 10% SDS-polyacrylamide gel electrophoresis (SDS-PAGE) and visualized by enhanced chemiluminescence according to the manufacturer’s instruction (Amersham). For immunoprecipitation assays, the lysates were mixed and precipitated with the relevant antibody and protein G-A agarose beads by overnight incubation at 4 °C. The bound proteins were resolved in 10% SDS-PAGE for immunoblot analysis.

### IKK kinase assay

Whole cell extracts were immunoprecipitated with an anti-IKKγ antibody, and protein A agarose beads by incubation at 4 °C for 4 h to overnight. The beads were washed with lysis buffer, and kinase assay was then performed in complete kinase assay buffer (20 mM HEPES at pH 7.5, 20 mM β-glycerol phosphate, 10 mM MgCl_2_, 1 mM DTT, 10 mM PNPP, 50 μM sodium vanadate, 20 μM ATP) with the addition of [γ-^32^P]-ATP and 1 μg of GST-IκBα (1-54) substrate. After 20 min at 30 °C, sample buffer was added and proteins were resolved in 12% SDS-polyacrylamide gels, and phosphorylated substrates were visualized by autoradiography.

### Luciferase reporter assay

293/IL-1R/TLR4 cells were co-transfected with p2xNF-κB-Luc, p2xTRE-Luc, pRSV-β-galactosidase, with or without of WT-Ref-1, ΔNLS-Ref-1 expression vector as indicated figure legends using Lipofectamine reagent according to the manufacturer’s instructions (Invitrogen). Twenty-four hours after transfection, cells were treated with LPS (1 μg/ml) for additional 10 h, and luciferase activities were measured using a luciferase assay kit (Promega, Madison, CA, USA). Luciferase activity was normalized relative to β-galactosidase activity of each sample.

### Determination of Intracellular ROS levels

The levels of intracellular ROS were measured using fluoresecent dye 2,7-dichlorofluorescein diacetate (DCF-DA). Cells were stained with 1 μM CM-H2DCFDA (Invitrogen Life Technologies, C6827) in Hank’s balanced salt solution for 30 min. Then cells were fixed with 4% paraformaldehyde before collecting cells. The stained cells were analyzed with FACSCanto II flow cytometer, and data were processed with the FlowJo software (FLOWJO).

### Assay for TNF-α secretion

TNF-α was measured by an enzyme linked immunosorbent assay (ELISA) according to the manufacturer’s instruction. Cells (2 x 10^5^ cells/well) were incubated for 24 h in medium alone or in medium containing LPS (1 μg/mL). The supernatants were collected and frozen at -80 °C until assayed for TNF-α.

### Statistical analysis

Numerical data are presented as mean ± SEM. Statistical significance of the data between groups was determined by using independent Student’s *t*-test. Analysis of variance (ANOVA), followed by post-hoc tests, was also used as needed.

## Abbreviations

AP-1: Activator protein 1
APE1: Apurinic/apyrimidinic endonuclease 1
ERK: Extracellular signal-regulated kinase
GFAP: Glial fibrillary acidic protein
IKK: IκB kinase
iNOS: Inducible nitric oxide synthase
JNK: c-jun N-terminal kinase
LPS: Lipopolysaccharide
NAC: N-acetyl-L-cysteine
NF-κB: Nuclear factor-κB
NLS: Nuclear import sequence
Ref-1: Redox effector factor-1
ROS: Reactive oxygen species
siRNA: Small interfering RNA
TNF-α: Tumor necrosis factor-α
